# Supplementary material for: Multi-Level Protocol for Mechanistic Reaction Studies Using Semi-Local Fitted Potential Energy Surfaces
Source: Int J Mol Sci. 2024 Aug 5;25(15):8530. doi: 10.3390/ijms25158530 (PMC11312657; doi:10.3390/ijms25158530)
Supplement: Supplementary file 1 [file ijms-25-08530-s001.zip › ijms-3105833-supplementary.pdf]

Supporting Information for: Multi-level Protocol for Mechanistic Reaction Studies Using Semi-local Fitted Potential Energy Surfaces

T. Piskor,*,†,‡ P. Pinski,† T. Mast,† and V. Rybkin†

†*HQS Quantum Simulations GmbH, Rintheimer Strasse 23, 76131 Karlsruhe, Germany*

‡*Theoretical Physics, Saarland University, 66123 Saarbrücken, Germany*

E-mail: tomislav.piskor@quantumsimulations.de,t.piskor@hotmail.de

1 Reaction rate coefficients

We use conventional transition state theory to compute rate coefficients for the two reactions as follows:[1]

$$k = \left(\frac{k_B T}{h}\right)\left(\frac{q_{trans,TS}/V}{q_{trans,R}/V}\right)\left(\frac{q_{rot,TS}/V}{q_{rot,R}/V}\right)\left(\frac{q_{vib,TS}/V}{q_{vib,R}/V}\right)e^{-\Delta E^{\ddagger}/k_B T}, \tag{1}$$

where k_B being the Boltzman constant, T - the temperature and h - the Planck constant. $q_{trans,TS}$, $q_{rot,TS}$ and $q_{vib,TS}$ denote the translational, rotational and vibrational partition functions for the transition state, whereas $q_{trans,R}$, $q_{rot,R}$ and $q_{vib,R}$ are their counterparts for the reactants.

The calculation of the rotational partition functions requires the eigenvalues of the inertia

tensor:

$$q_{rot}(T) = \frac{\pi^2}{\sigma} \sqrt{(8\pi I_A k_B T)/h^2} \sqrt{(8\pi I_B k_B T)/h^2} \sqrt{(8\pi I_C k_B T)/h^2}, \tag{2}$$

where I_A, I_B and I_C are the eigenvalues of the inertia tensor.

The harmonic vibrational partition function is defined as:

$$q_{vib}(T) = \prod_i^N \left(\frac{1}{1 - e^{\frac{-hc\nu_i}{k_B T}}} \right), \tag{3}$$

where ν_i are vibrational frequencies and N is the number of normal modes.

This translational partition function is defined as:

$$q_{trans}(V, T) = V \left(\frac{2\pi k_B T m}{h^2} \right)^{3/2}, \tag{4}$$

where m is the mass of the molecule.

For the unimolecular Bergman cyclization, the translational partition functions in equation 1 cancel out, whereas for the bimolecular S_N2 reaction they have to be included.

2 Harmonic vibrations

2.1 Vibrational modes for the Bergman cyclization of enediyne

Table S1: Comparison between CASSCF and sGDML frequencies for the reactant and transition state.

Mode number	ν^{R}_{CASSCF}/m^{-1}	ν^{R}_{sGDML}/m^{-1}	ν^{TS}_{CASSCF}/m^{-1}	ν^{TS}_{sGDML}/m^{-1}
0	10.3i	7.9i	799.5i	646.8i
1	7.2i	2.4i	0.9i	0.1i
2	0.7i	0.1i	0.0	0.0
3	0.1i	0.1i	0.4	0.0
4	2.9	0.3	3.5	7.2
5	6.3	3.0	7.7	9.0
6	100.5	102.7	8.1	11.2
7	211.7	206.7	348.1	353.1
8	220.4	225.0	422.5	403.4
9	323.1	324.8	485.2	491.0
10	402.5	422.9	543.8	502.1
11	564.7	548.1	598.4	528.5
12	577.9	567.8	639.9	617.9
13	578.1	574.4	679.2	665.1
14	591.1	581.0	736.2	677.5
15	594.1	589.8	818.2	835.3
16	728.9	725.9	856.7	949.5
17	778.1	742.7	975.7	956.7
18	900.5	883.0	1024.2	1015.0
19	1001.9	966.9	1033.1	1032.2
20	1063.1	1073.7	1148.2	1043.7
21	1291.9	1348.8	1210.1	1120.0
22	1497.3	1500.6	1453.9	1333.9
23	1658.1	1613.1	1479.7	1427.4
24	2238.8	2123.6	1713.0	1685.2
25	2246.3	2230.1	1885.0	1832.8
26	3338.3	3326.4	3365.9	3144.6
27	3354.6	3327.8	3381.2	3275.8
28	3611.0	3572.6	3482.0	3367.0
29	3611.2	3577.6	3487.3	3441.6

2.2 Vibrational modes for the SN2 reaction

Table S2: Comparison between CCSD and sGDML frequencies for the reactant and transition state.

Mode number	ν^{R}_{CCSD}/m^{-1}	ν^{R}_{sGDML}/m^{-1}	ν^{TS}_{CCSD}/m^{-1}	ν^{TS}_{sGDML}/m^{-1}
0	36.0i	3.2i	447.7i	448.1i
1	0.7i	1.4i	0.1i	0.3i
2	0.4i	0.7i	0.1i	0.2i
3	0.1	0.0	0.1	0.0
4	0.4	0.2	3.0	3.0
5	0.5	19.2	3.7	3.7
6	73.4	100.5	40.9	19.7
7	73.4	405.2	184.1	176.7
8	92.4	411.1	188.9	440.2
9	666.6	652.9	188.9	443.5
10	988.8	1022.0	921.5	1093.0
11	989.2	1030.7	922.1	1102.4
12	1328.1	1639.9	1003.4	1117.8
13	1468.1	1677.2	1409.1	1171.4
14	1468.4	1764.9	1409.2	1220.6
15	3152.2	2911.4	3228.6	2690.0
16	3275.1	3376.1	3440.0	3422.7
17	3275.6	3550.1	3441.8	3584.3

3 Reaction coordinate (NEB) for the S_N2 reaction

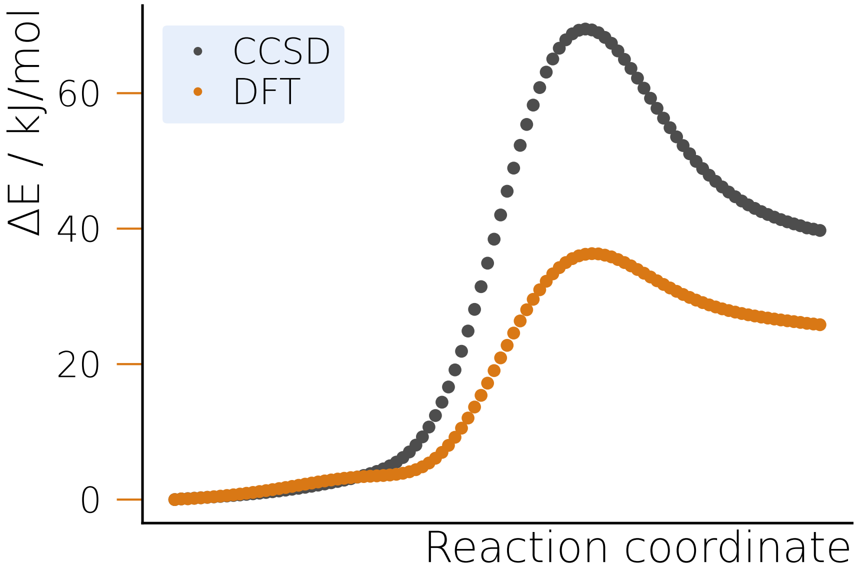

Figure S1: PES comparison between CCSD and DFT (PBE). Energies have been calculated for the same structures corresponding to the final state of the NEB calculation with DFT(PBE).

References

(1) Atkins, P.; Paula, J. *Atkins' physical chemistry*; Oxford University press, 2008.
